# *Pseudoalteromonas rhizosphaerae* sp. nov.*,* a novel plant growth-promoting bacterium with potential use in phytoremediation

**DOI:** 10.1099/ijsem.0.004167

**Published:** 2020-04-28

**Authors:** Salvadora Navarro-Torre, Lorena Carro, Ignacio D. Rodríguez-Llorente, Eloísa Pajuelo, Miguel Ángel Caviedes, José Mariano Igual, Hans-Peter Klenk, Maria del Carmen Montero-Calasanz

**Affiliations:** ^1^​ Departamento de Microbiología y Parasitología, Facultad de Farmacia, Universidad de Sevilla, Calle Profesor García González, 2 41012 Sevilla, Spain; ^2^​ Departamento de Microbiología y Genética. Universidad de Salamanca, 37007, Salamanca, Spain; ^3^​ Instituto de Recursos Naturales y Agrobiología de Salamanca, Consejo Superior de Investigaciones Científicas (IRNASA-CSIC), c/Cordel de Merinas 40-52, 37008 Salamanca, Spain; ^4^​ School of Natural and Environmental Sciences (SNES), Newcastle University, Newcastle upon Tyne, NE1 7RU, UK

**Keywords:** *Arthrocnemum macrostachyum*, heavy metals, odiel marshes, rhizosphere, nitrogen fixation

## Abstract

Strain RA15^T^ was isolated from the rhizosphere of the halophyte plant *Arthrocnemum macrostachyum* growing in the Odiel marshes (Huelva, Spain). RA15^T^ cells were Gram stain-negative, non-spore-forming, aerobic rods and formed cream-coloured, opaque, mucoid, viscous, convex, irregular colonies with an undulate margin. Optimal growth conditions were observed on tryptic soy agar (TSA) plates supplemented with 2.5 % NaCl (w/v) at pH 7.0 and 28 °C, although it was able to grow at 4–32 °C and at pH values of 5.0–9.0. The NaCl tolerance range was from 0 to 15 %. The major respiratory quinone was Q8 but Q9 was also present. The most abundant fatty acids were summed feature 3 (C_16 : 1_ **ω7*c* and/or C_16 : 1_ **ω6*c*), C_17 : 1_ *ω*8*c* and C_16 : 0_. The polar lipids profile comprised phosphatidylglycerol and phosphatidylethanolamine as the most abundant representatives. Phylogenetic analyses confirmed the well-supported affiliation of strain RA15^T^ within the genus *Pseudoalteromonas *, close to the type strains of *Pseudoalteromonas neustonica *, *Pseudoalteromonas prydzensis* and *Pseudoalteromonas mariniglutinosa *. Results of comparative phylogenetic and phenotypic studies between strain RA15^T^ and its closest related species suggest that RA15^T^ could be a new representative of the genus *Pseudoalteromonas *, for which the name *Pseudoalteromonas rhizosphaerae* sp. nov. is proposed. The type strain is RA15^T^ (=CECT 9079^T^=LMG 29860^T^). The whole genome has 5.3 Mb and the G+C content is 40.4 mol%.

*Pseudoalteromonas* [1] is the type genus of the family *Pseudoalteromonadaceae* [2]. It is currently shaped by 47 species [3] characterized by presenting Gram-stain-negative, motile, non-spore forming, aerobic, oxidase positive rods [4] requiring Na^+^ ions for growth [5] whose type species is *Pseudoalteromonas haloplanktis* [1, 4, 6]. Hitherto, all species described into the genus *Pseudoalteromonas* were isolated from marine environments, mainly seamount [7], sea water [4, 8–10], tidal flat [11] and as hosts of marine organisms [4, 12–14].

Strain RA15^T^ was isolated from the rhizosphere of *Arthrocnemum macrostachyum* plants growing in the Odiel marshes (Huelva, Spain) [15]. 16S rRNA gene sequence analysis showed that it clustered within the genus *Pseudoalteromonas* with 97.6 % similarity to the type strain of *Pseudoalteromonas prydzensis* [15]. It hydrolyses substrates such as starch, casein, Tween 80, DNA, chitin and pectin [15]. Furthermore, strain RA15^T^ demonstrated the ability to grow in high concentrations of heavy metals reaching values of 12 mM As and 3 mM Cu [15]. It also presented several plant growth-promoting (PGP) properties such as production of auxins and siderophores and nitrogen fixation, observing such traits even in presence of heavy metals, conditions under which higher expression of PGP traits were favoured [15]. Likewise, strain RA15^T^, as part of a bacterial consortium, improved the seed germination and the capacity of plants of *A. macrostachyum* to accumulate heavy metals in their roots [15, 16].

This report aims to elucidate the taxonomic status of strain RA15^T^, a strain showing biotechnological potential in the phytostabilization of heavy metal-polluted soils, in the genus *Pseudoalteromonas* following a polyphasic approach.

RA15^T^ was isolated from the rhizosphere of *A. macrostachyum* from the Odiel marshes (37° 13′ N, 6° 57′ W) as described in the work of Navarro-Torre *et al.* [15]. Succinctly, rhizosphere samples were mixed with sterile saline solution (0.9 %, w/v) and then shaken for 5 min. The suspension was plated on tryptic soy agar (TSA) plates supplemented with 2.25 % NaCl (w/v; salt concentration present in Odiel marshes) and incubated for 72 h at 28 °C. Different colonies were isolated according to morphology and colour criteria and sub-cultured. Pure cultures were preserved in 15 % glycerol at −80 °C.

Growth conditions were determined incubating the strain on TSA 2.25 % NaCl (w/v). The range of temperature for growth was tested at 4, 15, 20, 25, 28, 30, 32, 37 and 45 °C for 6 days. The range of pH for growth was assessed at pH 5.0, 6.0, 7.0, 8.0 and 9.0 for 6 days. pH values were adjusted with citrate–phosphate buffers (0.1M citric acid and 0.2M dibasic sodium phosphate) and Tris–HCl buffer (0.1M Tris (hydroxymethyl) aminomethane and 0.1M HCl). The growth in presence of NaCl was performed on mTGE (membrane tryptone glucose extract) agar medium [17] from 0 to 30 % (w/v) for 6 days at 28 °C. Growth under anaerobic conditions was carried out on semisolid TSA tubes containing 2.5 % NaCl (w/v) and sealed with a first layer of 2 % agar (w/v) and a second layer of paraffin and incubated for 10 days at 28 °C [18]. In addition, the bacterial growth in different media was determined on marine agar (MA) and the selective media cetrimide agar and MacConkey agar, both supplemented with 2.5 % NaCl (w/v), at 28 °C for 48 h.

Colony appearance was studied on TSA 2.5 % NaCl (w/v) plates after 24 h at 28 °C using a stereoscopic microscope (SZ61, Olympus). The colony colour was determined using the RAL D2 Design colour chart. Cell morphology was studied using an optical microscope with a ×100 objective (CX41, Olympus) after Gram staining [19]. Moreover cells of RA15^T^ were fixed with 2 % uranyl acetate and morphology was observed using a transmission electron microscope (Libra 120, Zeiss). Motility was tested by incubating the strain in tryptic soy broth (TSB) supplemented with 2.5 % NaCl (w/v) at 28 °C for 30 min [15]. After that, a drop from the culture was observed under optical microscope with a ×40 objective.

To determine the catalase activity, a drop of 3 % H_2_O_2_ was added to bacterial biomass. The test was considered positive if the reaction produced bubbles. For oxidase activity, 1 % *N*, *N*, *N*′, *N*′-tetramethyl-*p*-phenylenediamine reagent (Becton, Dickinson and Company) was added to bacterial biomass. When it turned blue in 10–15 s, the test was considered positive.

Biochemical characteristics were studied using API 20NE, API 20Strep and API ZYM galleries (bioMérieux) according to the manufacturer’s instructions. In addition, GEN III MicroPlates (Biolog) were used to determine the oxidation of carbon and nitrogen sources and the sensitivity to some inhibitory compounds. For the MicroPlate inoculations, strain RA15^T^ was resuspended in a viscous inoculating fluid (IF) A supplemented with 2.5 % NaCl (w/v) with a final transmittance of 95 % and then MicroPlates were inoculated. MicroPlates were incubated in an Omnilog device (Biolog) for 3 days at 30 °C. Results were analysed with the opm package for R version 1.3.72 [20, 21]. In parallel, the same protocol was carried out with the reference strains *Pseudoalteromonas prydzensis* DSM 14232^T^, *Pseudoalteromonas mariniglutinosa* DSM 15203^T^ and *Pseudoalteromonas neustonica* JCM 31286^T^.

Regarding chemotaxonomic analysis, studies of respiratory quinones, polar lipids and fatty acids were performed as follows: Respiratory quinones were extracted from freeze-dried biomass using aqueous methanol and petroleum ether [22]. Then, quinones were separated by thin-layer chromatography (TLC) in a chromatography tank containing petroleum ether and diethyleter (85 : 15; v/v) [22] and identified by HPLC [23]. Polar lipid extraction was also performed from freeze-dried biomass using aqueous methanol and petroleum ether [22] and then different polar lipids groups were separated using 2D-TLC [22]. For the detection of polar lipids, TLC plates were sprayed using molibdatophosphoric acid, ninhydrin, molybdenum blue and α-napthol [24, 25]. Finally, fatty acids extracted from 40 mg bacterial biomass grown on TSA supplemented with 2.5 % NaCl (w/v) for 24 h at 28 °C following the protocol outlined by Sasser [26]. Extracted fatty acids were identified using the Microbial Identification System (midi) Sherlock version 6.1 (RTSBA6 database). Fatty acids from the previously mentioned reference strains were also extracted in parallel experiments under the same growth conditions.

Genomic DNA was extracted using a G-spin Total DNA Extraction kit (Intron Biotechnology) according to the manufacturer’s instructions. The 16S rRNA gene was amplified as described in Navarro-Torre *et al.* [15]. The partial 16S rRNA gene sequence (1389 bp) was deposited in GenBank/EMBL/DDBJ data library under accession number KU588400 and aligned with corresponding sequences of closely related type strains retrieved by the Ez-Taxon-e service (www.ezbiocloud.net/eztaxon) [27]. 16S rRNA gene pairwise sequence similarities were determined using the method described by Meier-Kolthoff *et al.* [28]. The phylogenetic tree was inferred using the GGCD web server (http://ggdc.dsmz.de/) [29] according to Montero-Calasanz *et al.* [30]. The draft genome was sequenced using Illumina technology and a standard analysis pipeline by the MicrobesNG company (Birmingham, UK). The closest available reference was identified by Kraken [31]. Quality of data was estimated mapping the reads using BWA mem [32]. Then, *de novo* assembly was done with SPAdes [33] and again using BWA mem to get more quality metrics. The whole draft genome was deposited in GenBank/EMBL/DDBJ. Finally, the genome annotation and basic statistics were performed using the rast server version 2.0 [34], quast version 4.6.3 software [35], prokka [36], SignalP 4.1 server [37], TMHMM server version 2.0 [38] and CRISPRFinder [39]. Overall genome related indexes (OGRIs) were calculated using the GGCD web server [29] (http://ggdc.dsmz.de/) for the digital DNA–DNA hybridization (dDDH) test and the JSpeciesWS server [40] (http://jspecies.ribohost.com/jspeciesws) for the average nucleotide identity (ANI) test.

Cells of strain RA15^T^ were Gram-stain-negative, non-spore-forming, non-motile, aerobic rods of 2.1×1.3 µm (Fig. S1, available in the online version of this article). Cells appeared single or in pairs under optical microscope. Although most species described in the genus *Pseudoalteromonas* are motile [1, 4, 7, 8, 12, 41–43], the absence of motility is not exclusive of strain RA15^T^ as this characteristic was already noted in other species such as *Pseudoalteromonas gelatinilytica* [44]. RA15^T^ cells formed cream-coloured (RAL 075 90 20), opaque, mucoid, viscous, convex, irregular colonies with an undulate margin and were 3.75 mm in size after 24 h on TSA 2.5 % NaCl (w/v) plates at 28 °C. Strain RA15^T^ grew on TSA 2.5 % NaCl (w/v) in a range of temperature from 4 to 32 °C observing the optimal range from 20 to 28 °C. Range of pH was from 5.0 to 9.0 with an optimal pH at 7.0–8.0. The tolerance to NaCl was from 0 to 15 % (optimal growth at 2.5 %), but the growth in absence of NaCl was weak. These features were very similar to the other species of the genus and matched the genus description [1]. Strain RA15^T^ also was able to grow on MA as other species described in the genus [1, 4, 7, 8, 12, 44]. Contrarily, growth on MacConkey agar and cetrimide agar was not observed. According to Navarro-Torre *et al.* [15], strain RA15^T^ is positive for the hydrolysis of starch, casein, Tween 80, DNA, chitin and pectin. Here, positive results for gelatin and aesculin hydrolysis were also observed. The ability to hydrolyse both Tween 80 and gelatin is also in agreement with the emended description of the genus by Ivanova *et al.* [4]. Results from API ZYM, API 20NE and API 20Strep galleries reported the presence of alkaline phosphatase, esterase lipase (C8), leucine arylamidase, valine arylamidase, trypsine, acid phosphatase, naphthol-AS-BI-phosphosphohydrolase, *α*-glucosidase, *β*-glucosidase, *N*-acetyl-*β*-glucosaminadase, arginine dihydrolase, pyrolidonyl arylamidase and leucine aminopeptidase. Esterase (C4), cysteine arylamidase, *α*-chymotrypsin and *α*-galactosidase activity were also identified, but those results was weak. The presence of some enzymes like *β*-glucosidase, cysteine arylamidase, *α*-chymotrypsin, *α*-galactosidase, pyrolidonyl arylamidase and leucine aminopeptidase makes strain RA15^T^ metabolically different from other species of the genus *Pseudoalteromonas* [7, 8, 42, 43]. In addition, the API 20NE gallery showed that strain RA15^T^ reduced nitrates to nitrites and was able to assimilate d-glucose, l-arabinose, d-mannose, d-mannitol, *N*-acetyl-glucosamine, maltose, potassium gluconate, adipic acid and malic acid. Furthermore, RA15^T^ was Voges–Proskauer positive and produced the media acidification from d-ribose, trehalose, starch and glycogen according to API 20Strep gallery results. These results showed some differences from other species of the genus *Pseudoalteromonas* regarding the assimilation of adipic acid and the production of acetoin (Voges–Proskauer positive) [7, 8, 42, 43]. Finally, strain RA15^T^ was catalase- and oxidase-positive in line with other species in the genus [1]. Concerning results from the Biolog system, strain RA15^T^ was able to oxidise dextrin, maltose, trehalose, cellobiose, *β*-gentiobiose, sucrose, *N*-acetyl-d-glucosamine, *N*-acetyl-*β*-d-mannosamine, *N*-acetyl-d-galactosamine, d-glucose, d-fructose, d-galactose, l-fucose, l-rhamnose, inosine, 1 % sodium lactate, fusidic acid, d-serine, d-mannitol, d-glucose-6-phosphate, d-fructose-6-phosphate, gelatin, glycine-proline, l-alanine, l-arginine, l-aspartic acid, l-glutamic acid, l-histidine, l-serine, pectin, d-galacturonic acid, l-galactonic acid-*γ*-lactone, d-gluconic acid, l-malic acid, Tween 40, *α*-keto-butyric acid, acetoacetic acid, propionic acid and acetic acid. Moreover, strain RA15^T^ tolerated the presence of rifamycin SV, tetrazolium violet, tetrazolium blue and potassium tellurite. Strain RA15^T^ showed features in common with the closely related species but also some differences (a summary of differential physiological characteristics shown between strain RA15^T^ and its closely related type strains is provided in Table 1; full Biolog GEN III MicroPlate system results are provided in Table S1).

**Table 1.** Differential phenotypic characteristics between strain RA15^T^ and related species of the genus *Pseudoalteromonas
* Strains; 1, RA15^T^; 2, *Pseudoalteromonas prydzensis* DSM 14232^T^; 3, *Pseudoalteromonas mariniglutinosa* DSM 15203^T^; 4, *Pseudoalteromonas neustonica* JCM 31286^T^. +, Positive; −, negative; w, weak; nd, data no available. Data are taken from this study unless indicated. According to the GEN III system, all strains were positive for dextrin, gentiobiose, pH 6, 1 % NaCl, 4 % NaCl, d-glucose, 1 % sodium lactate, pectin, d-galacturonic acid, d-fructose-6-phosphate, l-galactonic acid-γ-lactone and potassium tellurite; all strains were negative for turanose, stachyose, lactose, d-arabitol, myo-inositol, d-sorbitol, d-aspartic acid, glycerol, lincomycin, guanidine hydrochloride, quinic acid, vancomycin, α-keto-glutaric acid, d-malic acid, γ-amino-n-butyric acid and sodium formate.

Characteristics	1	2	3	4
NaCl range for growth (%, w/v)	0–15	0.5–15*	1–9†	1–7‡
Temperature range for growth (°C)	4–32	0–30*	5–37†	4–30‡
Optimal temperature for growth (°C)	20–28	22–25	20–28†	25‡
Hydrolysis of:				
Aesculin	+	+*	+†	−‡
Enzymatic activity according to API ZYM:				
Esterase lipase (C8)	+	nd	w†	−‡
Valine arylamidase	+	nd	−†	−‡
Trypsine	+	nd	−†	−‡
*α*-Glucosidase	+	nd	−†	−‡
Arginine dihydrolase	+	−*	−†	−‡
Reduction nitrates to nitrites (API 20NE)	+	−*	−†	−‡
According to GEN III system, it oxidises:				
Maltose	+	+	+	−
Sucrose	+	+	+	−
Melibiose	−	+	−	+
Methyl *β*-d-glucoside	−	+	−	+
*N*-acetyl-d-glucosamine	+	+	+	−
*N*-acetyl-neuraminic acid	−	−	−	+
d-Fructose	+	+	−	−
d-Galactose	+	−	−	+
3-*O*-Methyl-d-glucose	−	+	−	+
l-Fucose	+	−	−	+
l-Rhamnose	+	−	+	+
d-Glucose-6-phosphate	+	+	+	−
Troleandomycin	−	−	−	+
l-Alanine	+	+	+	−
l-Arginine	+	+	+	−
d-Glucuronic acid	−	+	+	+
Tetrazolium blue	+	−	−	+

*Data from Bowman *et al.* [41].

†Data from Romanenko *et al.* [42].

‡Data from Hwang *et al.* [8].

The major respiratory quinones were Q8 (65.8 %) and Q9 (11.6 %) in keeping with data for other species of *Pseudoalteromonas* [7, 43, 44] and according with the family description [2]. The polar lipid profile consisted of phosphatidylglycerol and phosphatidylethanolamine (Fig. 1), as in other species of the genus [7, 42, 44], and two unidentified aminolipids. The major fatty acids were summed feature 3 (C_16 : 1_ ω7*c* and/or C_16 : 1_ **ω6*c*; 35.3%), C_17 : 1_ *ω*8*c* (16.7 %) and C_16 : 0_ (12.2 %) (Table 2).

**Fig. 1.**
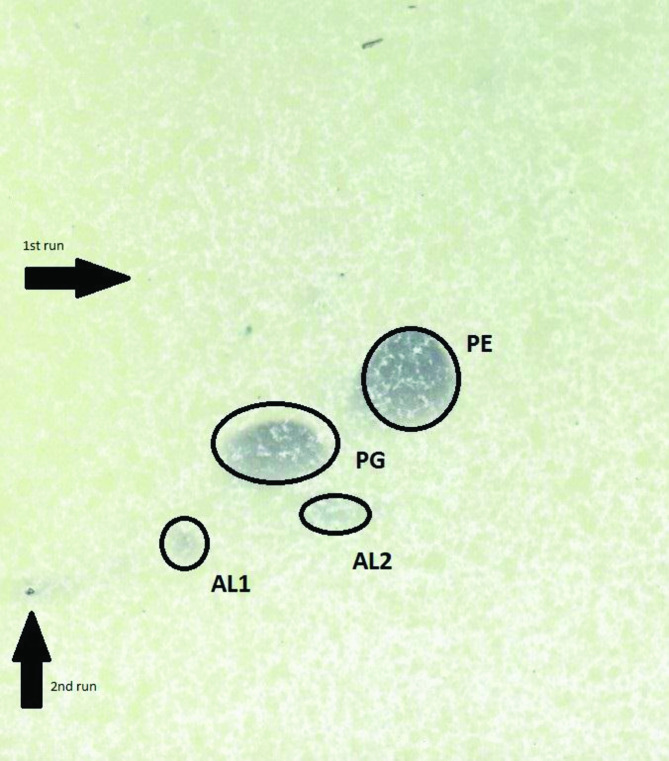
Total lipid profiles (labelled by the Rf values) of strain RA15^T^ after separation by two-dimensional TLC using the solvents chloroform/methanol/water (65 : 25 : 4, by vol.) in the first dimension and chloroform/methanol/acetic acid/water (80 : 12 : 15 : 4, by vol.) in the second dimension. Plates were sprayed with molybdatophosphoric acid (3.5 %; Merck) for detection of total polar lipids. PE, phosphatidylethanolamine; PG, phosphatidylglycerol; AL1–2, unidentified aminolipids.

**Table 2.** Cellular fatty acid compositions (%) of RA15^T^ and closely related *Pseudoalteromonas* species Strains: 1, RA15^T^; 2, *Pseudoalteromonas prydzensis* DSM 14232^T^; 3, *Pseudoalteromonas mariniglutinosa* DSM 15203^T^; 4, *Pseudoalteromonas neustonica* JCM 31286^T^. –, Not detected; tr, values below 1 %. Values below 1 % in all columns are not displayed. All data are obtained in this study.

Fatty acid	1	2	3	4
C_11 : 0_ 3-OH	2.4	1.1	2.2	–
C_12 : 0_	2.7	2.2	2.3	tr
iso-C_12 : 0_ 3-OH	1.7	1.6	1.9	–
C_12 : 0_ 3-OH	5.2	6.3	6.4	–
C_14 : 0_	1.0	2.2	1.5	5.6
C_15 : 1_ *ω*8*c*	4.7	3.4	2.5	tr
Summed feature 3*§	35.3	38.7	33.3	–
C_16 : 0_	12.2	20.8	16.7	46.2
iso-C_16 : 0_	tr	1.6	1.3	–
C_17 : 1_ *ω*8	16.7	7.9	11.8	–
anteiso-C_17 : 0_	tr	1.2	1.2	–
C_17 : 0_	4.5	2.7	3.7	–
C_18 : 0_	tr	tr	1.1	1.4
C_18 : 1_ *ω*9*c*	tr	tr	1.4	38.5
10-Methyl C_18 : 0_	–	–	–	4.6
Summed feature 8†§	6.4	4.7	4.7	–
Summed feature 7‡§	tr	tr	4.7	tr

*Summed feature 3 was listed as C_16 : 1_ ω7*c* and/or C_16 : 1_ ω6*c*.

†Summed feature 8 was listed as C_18 : 1_ ω7*c* and/or C_18 : 1_ ω6*c*.

‡Summed feature 7 was listed as C_19 : 1_ ω7*c* and/or C_19 : 1_ ω6*c*

§Summed features are groups of two or three fatty acids that are treated together for the purpose of evaluation in the midi system and include both peaks with discrete equivalent chain lengths (ECLs) as well as those where the ECLs are not reported separately [46].

Phylogenetic analyses affiliated strain RA15^T^ to the genus *Pseudoalteromonas* forming a well-supported group with the type strains of *P. neustonica, P. prydzensis* and *P. mariniglutinosa* (Fig. 2). 16S rRNA gene sequence similarities to *P. neustonica* JCM 31286^T^ (98.5 %), *P. prydzensis* MB8-11^T^ (98.1 %) and *P. mariniglutinosa* KMM 3635^T^ (97.8 %) were nevertheless below the 98.7 % threshold recommended [28, 45] to confirm species novelty in the phylum *Proteobacteria *. Results of OGRI tests performed with the draft genomes of *P. neustonica* PAMC 28425^T^ (accession number BDDS01), *P. prydzensis* MB8-11^T^ (accession number BDDT01) and *P. mariniglutinosa* KMM 3635^T^ (accession number BDDU01; Table 3) supported the taxonomic allocation of strain RA15^T^ as new representative of the genus *Pseudoalteromonas* [28, 45].

**Fig. 2.**
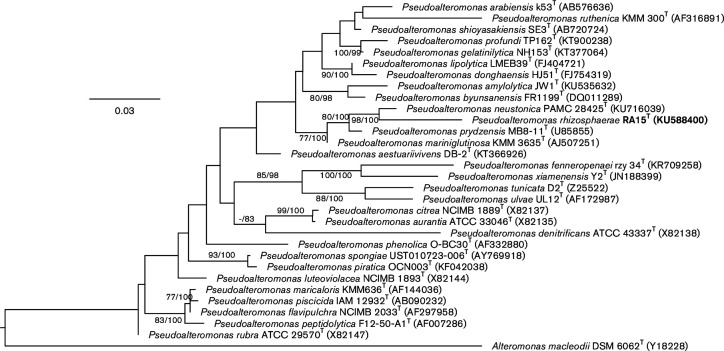
Maximum-likelihood phylogenetic tree inferred from 16S rRNA gene sequences, showing the phylogenetic position of strain RA15^T^ relative to type strains of species within the genus *Pseudoalteromonas *. The branches are scaled in terms of the expected number of substitutions per site. Support values obtained from 1000 replicates from maximum-likelihood (left) and maximum-parsimony (right) bootstrapping are shown above the branches if ≥60 %. Sequence accession numbers are given in parentheses.

**Table 3.** Overall genome related index (OGRI) of strain RA15^T^

Strains	dDDH	ANIb	ANIm
* Pseudoalteromonas neustonica* PAMC 28425^T^	24.50 %	80.54 %	85.45 %
* Pseudoalteromonas prydzensis* MB8-11^T^	28.20 %	83.93 %	86.78 %
* Pseudoalteromonas mariniglutinosa* KMM 3635^T^	25.80 %	82.11 %	85.45 %

Following the proposed minimal standards for the use of genome data for the taxonomy of prokaryotes [45], the whole genome sequence of strain RA15^T^, whose accession number is CABVLM01, has a total length of 5,267,131 bp, and is formed of 97 contigs. The N50 value is 328, 874, the coverage is 36.7× and the genomic G+C content of 40.4 mol% (Table S2).

Combined phenotypic and phylogenetic data demonstrated that RA15^T^ represents a novel specie in the genus *Pseudoalteromonas *, and the name *Pseudoalteromonas rhizosphaerae* sp. nov. is proposed.

## Description of *Pseudoalteromonas rhizosphaerae* sp. nov.

*Pseudoalteromonas rhizosphaerae* (rhi.zo.sphae′rae. N.L. gen. n. *rhizosphaerae* of the rhizosphere).

Cells are Gram-stain-negative, non-spore-forming, aerobic rods appearing alone or in pairs. Cells form cream-coloured (RAL 075 90 20), opaque, mucoid, viscous, convex, irregular colonies with an undulate margin of 3.75 mm after 24 h on TSA 2.5 % NaCl (w/v) plates at 28 °C (optimal growth conditions). It grows at 4–32 °C and pH 5.0–9.0 and tolerates 0–15 % NaCl (w/v). Grows well on MA, but not on MacConkey agar or cetrimide agar. Catalase- and oxidase-positive. Starch, casein, Tween 80, DNA, chitin, pectin, gelatin and aesculin are hydrolysed. According to API ZYM assay results, strong enzymatic activity is observed for alkaline phosphatase, esterase lipase (C8), leucine arylamidase, valine arylamidase, trypsine, acid phosphatase, naphthol-AS-BI-phosphosphohydrolase, *α*-glucosidase, *β*-glucosidase, *N*-acetyl-*β*-glucosaminadase, arginine dihydrolase, pyrolidonyl arylamidase and leucine aminopeptidase; weak for esterase (C4), cysteine arylamidase, *α*-chymotrypsin and *α*-galactosidase, and negative for alkaline phosphatase, lipase (C14), *β*-galactosidase, *β*-glucoronidase, *α*-mannosidase and *α*-fucosidase. According API 20NE, can reduce nitrates to nitrites and is able to assimilated-glucose, _L_-arabinose, d-mannose, d-mannitol, *N*-acetyl-glucosamine, maltose, potassium gluconate, adipic acid and malic acid, but cannot assimilate capric acid, trisodium citrate and phenylacetic acid, ferment glucose or produce indole. Positive for production of acetone (Voges–Proskauer positive). Acid is formed from d-ribose, trehalose, starch and glycogen, but not from l-arabinose, d-mannitol, d-sorbitol, lactose, inulin and raffinose. According to the Biolog system, positive for the oxidation of dextrin, maltose, trehalose, cellobiose, *β*-gentiobiose, sucrose, *N*-acetyl-d-glucosamine, *N*-acetyl-*β*-d-mannosamine, *N*-acetyl-d-galactosamine, d-glucose, d-fructose, d-galactose, l-fucose, l-rhamnose, inosine, 1 % sodium lactate, fusidic acid, d-serine, d-mannitol, d-glucose-6-phosphate, d-fructose-6-phosphate, gelatin, glycine-proline, l-alanine, l-arginine, l-aspartic acid, l-glutamic acid, l-histidine, l-serine, pectin, d-galacturonic acid, l-galactonic acid-*γ*-lactone, d-gluconic acid, l-malic acid, Tween 40, *α*-keto-butyric acid, acetoacetic acid, propionic acid and acetic acid, but negative for turanose, stachyose, raffinose, lactose, melibiose, methyl *β*-d-glucoside, d-salicin, *N*-acetyl-neuraminic acid, d-mannose, 3-*O*-methyl-d-glucose, d-fucose, d-sorbitol, d-arabitol, myo-inositol, glycerol, d-aspartic acid, troleandomycin, minocycline, l-pyroglutamic acid, guanidine hydrochloride, d-glucuronic acid, glucuronamide, mucic acid, quinic acid, d-saccharic acid, *p*-hydroxy-phenylacetic acid, methyl pyruvate, d-lactic acid methyl ester, l-lactic acid, citric acid, *α*-keto-glutaric acid, d-malic acid, bromo-succinic acid, *γ*-amino-*n*-butyric acid, *α*-hydroxy-butyric acid, *β*-hydroxy-butyric acid, sodium formate, butyric acid and sodium bromate. It respirates in presence of rifamycin SV, tetrazolium violet, tetrazolium blue and potassium tellurite but not in the presence of aztreonam, nalidixic acid, lithium chloride vancomycin lincomycin and niaproof. The major fatty acids are summed feature 3 (C_16 : 1_ ω7c and/or C_16 : 1_ ω6*c*), C_17 : 1_ ω8*c* and C_16 : 0_. The predominant respiratory quinones are Q8 and Q9. The polar lipid profile consists of phosphatidylglycerol, phosphatidylethanolamine and two unidentified aminolipids. The whole genome has a total length of 5,267, 131 bp and is formed of 97 contigs. The N50 value is 328,874 and the coverage is 36.7×. The genomic G+C content is 40.4 mol%.

The type strain, RA15^T^ (=CECT 9079^T^=LMG 29860^T^), was isolated from the rhizosphere of the halophyte plant *Arthrocnemum macrostachyum*. The GenBank/EMBL/DDBJ accession number for the 16S rRNA gene sequence is KU588400. The GenBank/EMBL/DDBJ accession number for the draft genome is CABVLM01.

## Supplementary Data

Supplementary material 1
