# Supplementary material for: Pseudoalteromonas rhizosphaerae sp. nov., a novel plant growth-promoting bacterium with potential use in phytoremediation
Source: Int J Syst Evol Microbiol. 2020 Apr 28;70(5):3287–94. doi: 10.1099/ijsem.0.004167 (PMC7395622; doi:10.1099/ijsem.0.004167)
Supplement: Supplementary material 1 [file ijsem-70-3287-s001.pdf]

International Journal of Systematic and Evolutionary Microbiology

Supplementary Material

Pseudoalteromonas rhizosphaerae sp. nov., a novel plant growth-promoting bacterium with potential use in phytoremediation.

Salvadora Navarro-Torre[1], Lorena Carro[2], Ignacio D. Rodríguez-Llorente[1], Eloísa Pajuelo[1], Miguel Ángel Caviedes[1], José Mariano Igual[3], Hans-Peter Klenk[4] & Maria del Carmen Montero-Calasanz[4*]

[1]Departamento de Microbiología y Parasitología, Facultad de Farmacia, Universidad de Sevilla, Calle Profesor García González, 2, 41012 Sevilla, Spain.

[2]Departamento de Microbiología y Genética. Universidad de Salamanca, 37007, Salamanca, Spain.

[3]Instituto de Recursos Naturales y Agrobiología de Salamanca, Consejo Superior de Investigaciones Científicas (IRNASA-CSIC), c/Cordel de Merinas 40-52, 37008 Salamanca, Spain.

[4]School of Natural and Environmental Sciences (SNES), Newcastle University, Newcastle upon Tyne, NE1 7RU, UK.

***Corresponding author:** Maria del Carmen Montero-Calasanz Tel.: +44 (0)191.208.4943 e-mail: maria.montero-calasanz@ncl.ac.uk

Running title: *Pseudoalteromonas rhizosphaerae* sp. nov.

Supplementary Figure S1. Scanning electron micrograph of strain RA15^{T} grown on TSA 2.5% NaCl medium for 24 hours at 28°C.

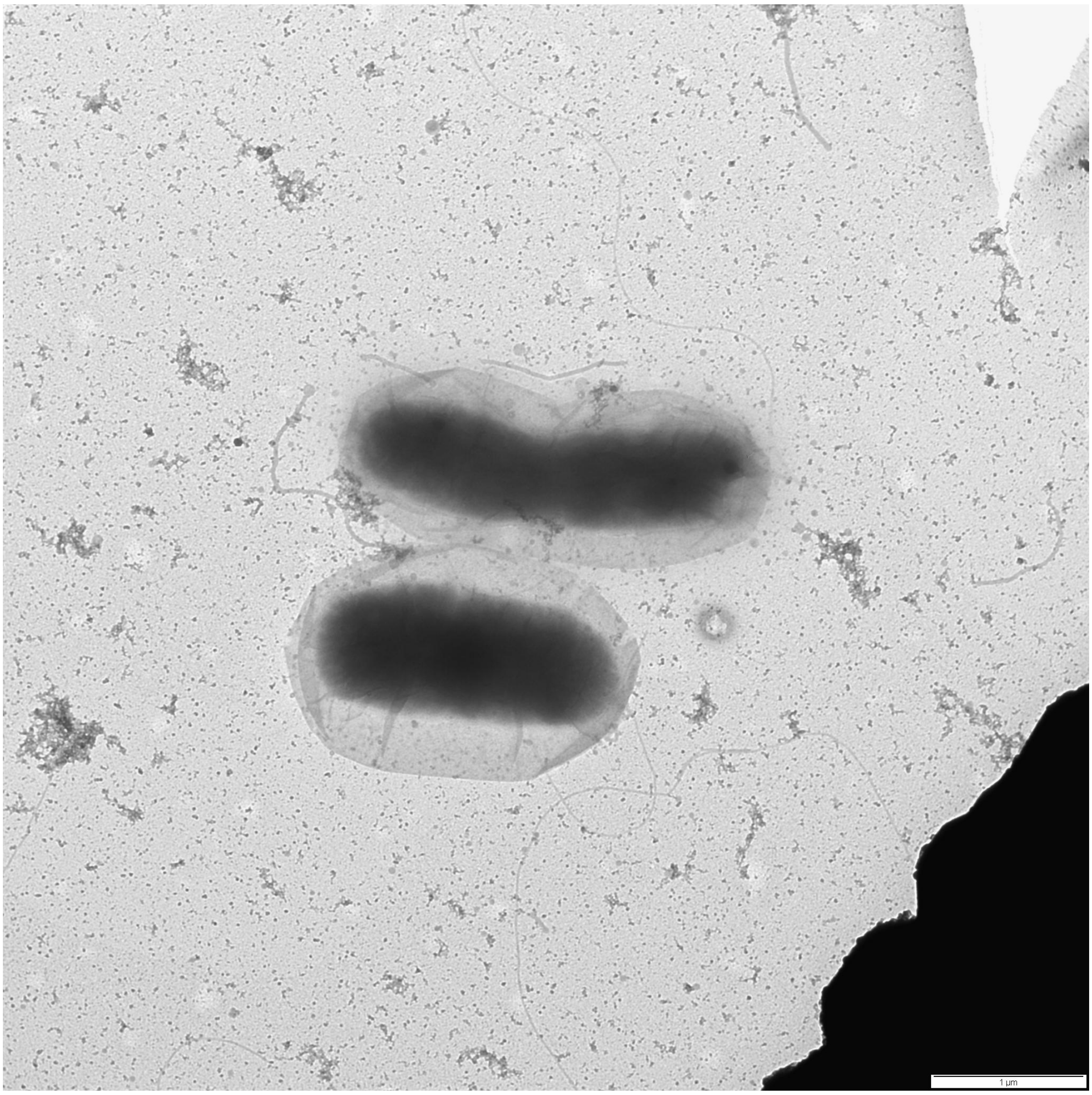

Supplementary Table S1. Phenotypic characteristics of strain RA15^T and related species of the genus *Pseudoalteromonas* according to GEN III Microplates system.

Strains; 1, Strain RA15^T; 2, *P. prydzensis* DSM 14232^T; 3, *P. mariniglutinosa* DSM 15203^T; 4, *P. neustonica* JCM 31286^T. +, positive; -, negative ; +/-, ambiguous; Data are taken from this study. Sub-inhibitory and inhibitory concentrations of serine are indicated as #1 and #2, respectively.

**Characteristic**	**1**	**2**	**3**	**4**
Dextrin	+	+	+	+
D-Maltose	+	+	+	-
D-Trehalose	+	+	-	+/-
D-Cellobiose	+	+	+	+/-
β-Gentiobiose	+	+	+	+
Sucrose	+	+	+	-
Turanose	-	-	-	-
Stachyose	-	-	-	-
pH 6	+	+	+	+
pH 5	-	-	+	+/
D-Raffinose	-	+	+	+/
α-D-Lactose	-	-	-	-
D-Melibiose	-	+	-	+
β-Methyl-D-Glucoside	-	+	-	+
D-Salicin	-	-	-	+/-
N-Acetyl-D-Glucosamine	+	+	+	-
N-Acetyl-*β*-D-Mannosamine	+	+	-	+/
N-Acetyl-D-Galactosamine	+	+	-	+/
N-Acetyl-Neuraminic Acid	-	-	-	+
1% NaCl	+	+	+	+
4% NaCl	+	+	+	+
8% NaCl	+	+	+	+/-
D-Glucose	+	+	+	+
D-Mannose	-	-	-	+/-
D-Fructose	+	+	-	-
D-Galactose	+	-	-	+
3-*O*-Methyl-D-Glucose	-	+	-	+
D-Fucose	-	+	+	+/-
L-Fucose	+	-	-	+
L-Rhamnose	+	-	+	+
Inosine	+	+	+	+/-
1% Sodium Lactate	+	+	+	+

Fusidic Acid	+	-	+	+/
D-Serine #2	+	-	+	+/
D-Sorbitol	-	-	-	-
D-Mannitol	+	-	+	+/-
D-Arabitol	-	-	-	-
myo-Inositol	-	-	-	-
Glycerol	-	-	-	-
D-Glucose-6-Phosphate	+	+	+	-
D-Fructose-6-Phosphate	+	+	+	+
D-Aspartic Acid	-	-	-	-
D-Serine #1	-	-	+	+
Troleandomycin	-	-	-	+
Rifamycin SV	+	-	-	+/-
Minocycline	-	-	-	+
Gelatin	+	+	+	+/-
Glycine-Proline	+	+	+	-
L-Alanine	+	+	+	-
L-Arginine	+	+	+	-
L-Aspartic Acid	+	+	+	-
L-Glutamic Acid	+	+	+	+/-
L-Histidine	+	+	+	-
L-Pyroglutamic Acid	-	-	-	+/-
L-Serine	+	+	+	-
Lincomycin	-	-	-	-
Guanidine Hydrochloride	-	-	-	+
Niaproof	-	-	-	+/-
Pectin	+	+	+	+
D-Galacturonic Acid	+	+	+	+
L-Galactonic Acid-γ-Lactone	+	+	+	+
D-Gluconic Acid	+	+	-	+/-
D-Glucuronic Acid	-	+	+	+
Glucuronamide	-	-	-	+
Mucic Acid	-	+	-	-
Quinic Acid	-	-	-	-
D-Saccharic Acid	-	-	-	+/-
Vancomycin	-	-	-	-
Tetrazolium Violet	+	-	-	+
Tetrazolium Blue	+	-	-	+
p-Hydroxy-Phenylacetic Acid	-	-	-	+
Methyl Pyruvate	-	+	-	-
D-Lactic Acid Methyl Ester	-	+	-	+/-

L-Lactic Acid	-	-	+	-
Citric Acid	-	-	-	+/-
α-Keto-Glutaric Acid	-	-	-	-
D-Malic Acid	-	-	-	-
L-Malic Acid	+	+	+	-
Bromo-Succinic Acid	-	-	-	+/-
Nalidixic Acid	-	-	+	+
Lithium Chloride	-	-	+	+
Potassium Tellurite	+	+	+	+
Tween 40	+	+	-	+/-
γ-Amino-*n*-Butyric Acid	-	-	-	-
α-Hydroxy-Butyric Acid	-	-	+	-
β-Hydroxy-Butyric Acid	-	+	-	+/-
α-Keto-Butyric Acid	+	+	+	-
Acetoacetic Acid	+	-	+	+
Propionic Acid	+	+	+	+/-
Acetic Acid	+	+	+	-
Sodium Formate	-	-	-	-
Aztreonam	-	-	+	+
Butyric Acid	-	-	+	+
Sodium Bromate	-	-	-	+

Supplementary Table S2. Genome statistics.

Attribute	Value
Genome size (bp)[a]	5,267,131
DNA G + C (%)[a]	40.4
Number of contigs[a]	97
Largest contig[b]	730,648
Number of CDS[c]	4,565
RNA genes[c]	109
tmRNA[c]	1
rRNA[c]	9
tRNA[c]	99
N50[a]	328,874
L50[a]	6
N75[b]	170,293
L75[b]	12
Genes with signal peptides[d]	898
Genes with transmembrane helices[e]	1154
CRISPR repeats[f]	3
Total number of chromosomes and plasmids	1

[a] Data from RAST v2.0 [34].
[b] Data from QUAST v.4.6.3 software [35].
[c] Data from Prokka [36].
[d] Data from SignalP 4.1 server [37].
[e] Data from TMHMM server v.2.0 [38].
[f] Data from CRISPRFinder [39].
